# *Mycobacterium ulcerans*-*Bordetella trematum* chronic tropical cutaneous ulcer: A four-case series, Côte d’Ivoire

**DOI:** 10.1371/journal.pntd.0011413

**Published:** 2023-12-07

**Authors:** Bi Goré Oscar Tchan, Solange Kakou-Ngazoa, Sylveste Dizoe, Nassim Hammoudi, Ghiles Grine, Raymond Ruimy, Michel Drancourt

**Affiliations:** 1 Aix-Marseille-Université, IRD, MEPHI, IHU Méditerranée Infection, Marseille, France; 2 IHU Méditerranée Infection, Marseille, France; 3 Plateforme de biologie moléculaire, Institut Pasteur de Côte d’Ivoire, Abidjan, Côte d’Ivoire; 4 National Buruli ulcer Control Program, Abidjan, Côte d’Ivoire; 5 Department of Bacteriology, Nice Academic Hospital, Nice, France; 6 Université Côte D’Azur, CHU de Nice, Nice, France; International Foundation for Dermatology, London, United Kingdom, UNITED KINGDOM

## Abstract

**Background:**

Chronic tropical cutaneous ulcers remain a neglected medical condition in West Africa, particularly Buruli ulcer, which is caused by mycolactone cytotoxin-secreting *Mycobacterium ulcerans* (*M*. *ulcerans*). Medical management of this highly debilitating and necrotising skin infection may be modified by colonisation and co-infection of the ulcer by opportunistic and pathogenic microorganisms, which considerably delays and increases the cost of treatment.

**Methodology/principal finding:**

We diagnosed chronic tropical cutaneous ulcers in nine patients in Côte d’Ivoire using *M*. *ulcerans*-specific PCRs and culturomics. This revealed *M*. *ulcerans* in 7/9 ulcer swabs and 5/9 control swabs as well as an additional 122 bacterial species, 32 of which were specific to ulcers, 61 specifics to the controls, and 29 which were shared, adding 40 bacterial species to those previously reported. Whole genome sequencing of four *Bordetella trematum* (*B*. *trematum*) isolates in four Buruli ulcer swabs and no controls indicated cytolethal distending toxins, as confirmed by cytotoxic assay.

**Conclusions/significance:**

In four cases of Buruli ulcer in Côte d’Ivoire, *B*. *trematum* was a co-pathogen which was resistant to rifampicin and clarithromycin, unmatching *M*. *ulcerans* antibiotic susceptibility profile and counteracting the current treatment of Buruli ulcer in West Africa and Australia. Thus, we report here chronic mixed *M*. *ulcerans*-*B*. *trematum* chronic tropical ulcer as a specific form of Buruli ulcer in West Africa.

## Author summary

Buruli ulcer, a severe necrotising skin infection caused by *M*. *ulcerans*, remains a neglected health problem in some rural communities in West Africa. Although it can be successfully treated with an antibiotic combination of rifampicin and streptomycin or clarithromycin, the presence of other pathogens or opportunistic agents complicates its management. These co-infections or superinfections make treatment more complex and expensive. In this study, we highlight the association of *M*. *ulcerans*, a cytotoxin producer, with *B*. *trematum*, another toxin producer associated with chronic ulcers in Buruli ulcer patients. *B*. *trematum* shows resistance to the antibiotics used in the Buruli ulcer treatment regimen. Therefore, it is essential to conduct further research to understand the magnitude of its contribution to the illness.

## Introduction

Tropical cutaneous chronic ulcers cause stigmatisation and are a significant handicap for the rural populations which mostly suffer from this this disabling condition. Nonetheless, it remains a neglected health problem in some West African countries [1]. Several neglected tropical diseases (NTDs) are known to cause tropical cutaneous chronic ulcers including Buruli ulcer, yaws, leprosy, scabies and advanced lymphatic filariasis [2]. Of these, Buruli ulcer is a necrotising and debilitating skin disease caused by *Mycobacterium ulcerans* (*M*. *ulcerans*) and reported by 34 countries, with West Africa and Australia the most affected [3]. The disease progresses from cutaneous nodules to papules and oedema, eventually forming an ulcer with undermined edges [4]. Further superinfection by pathogenic and opportunistic bacteria issued from the normal skin flora or the immediate environment, mainly *Staphylococcus aureus* (*S*. *aureus*) and *Pseudomonas aeruginosa* (*P*. *aeruginosa*), makes Buruli ulcer treatment challenging [5,6]. These pathogens are further associated with delays in the healing process and significant increases in treatment costs [5–7].

In this study we investigate a series of collected swabs samples from patients in the *M*. *ulcerans*-endemic region of Yamoussoukro, Côte d’Ivoire [8–9]. Patients presented with tropical cutaneous chronic ulcer clinically diagnosed as Buruli ulcer. The results unexpectedly revealed a toxigenic *Bordetella trematum* (*B*. *trematum*) infection mixed with *M*. *ulcerans* in some patients, leading to the description of tropical cutaneous mixed bordetellomatosis-mycobacteriosis as a new clinical entity to be considered as a specific form of Buruli ulcer in West Africa. This also results in a complete, unprecedented characterisation of chronic cutaneous ulcer-associated *B*. *trematum* isolates.

## Patients and methods

### Ethics statement

The study included patients (and parent in the case of infant) who gave oral consent for swabbing healthy skin. Accordingly, the study involved a non-invasive procedure and was approved by the National Committee for the Ethics of Life Sciences and Health of Côte d’Ivoire under the reference 19-22/MSHP/CNESVS.

### Patients

Nine patients consulted for chronic cutaneous ulcer as part of the routine medical practice at one of the authors’ (SD) Buruli ulcer medical management centres in the Yamoussoukro Health District in Côte d’Ivoire. These nine patients lived in the city of Yamoussoukro or surrounding rural areas in the Bélier district, an area that has been acknowledged for decades as being endemic for Buruli ulcer [9]. Patient demographics and ulcer history are reported in Table 1. For each cutaneous ulcer, one swab contained in a transport medium (∑-Transwab, International Medical Products, Belgium) and one dry swab were gently applied by SD to the ulcer to perform routine and advanced microbiological investigations, seeking the microbial aetiology of the ulcer. With the oral consent of each patient, similar non-invasive swabbing was then performed on the skin surface contralateral to the ulcer, as an auto control clinical specimen. Collected swabs were anonymously coded, divided into two batches and placed in a well-sealed cooler containing dry ice. One batch was sent to the Institut Pasteur in Côte d’Ivoire for first line diagnosis and the other batch was shipped at ambient temperature to the IHU-Méditerranée Infection in Marseille, France for advanced diagnosis.

**Table 1:** Patient demographics. Timescale refers to delay for ulcer evolution. NA, not available.

Patient’s code	Age range (years)	Gender	Localisation	Timescale (months)
CI016	[10–19]	Male	Left ankle	8
CI014	[10–19]	Male	Left thigh	6
CI027	[20–29]	Male	Right tibia	NA
CI032	[30–39]	Male	Right hand	180
CI040	[40–49]	Male	Left elbow	NA
CI053	[50–59]	Female	Righr tibia	1
CI062	[60–69]	Female	External face of left calf	60
CI070	[70–79]	Male	Right foot	3
CI083	[80–89]	Male	Medial malleolus right foot	1.5

### Molecular detection of *M*. *ulcerans*

Total DNA from all samples (lesion and auto control samples) was extracted using the QIAMP tissue kit on a QIAGEN-BioRobot EZ1 according to the manufacturer’s instructions (Qiagen, Hilden, Germany). Extracted DNA was incorporated using real-time PCR (RT-PCR) to amplify the insertion sequences (IS2404 and IS2606) and the ketoreductase-B domain of the mycolactone polyketide synthase (KR-B) gene, using the RT-PCR reagents of the Roche PCR kit (Roche Diagnostics, Meylan, France) and the primers and probes previously described [10], in the CFX 96 real-time PCR detection and thermal cycler system (Bio-Rad, Marnes-la-Coquette, France) in the presence of negative controls. Samples were considered positive when the KR-B gene RT-PCR yielded a Ct < 40 cycles and at least one of the two insertion sequence PCRs yielded a Ct < 40 cycles, as previously described [11].

### Swab culturomics

One hundred microliters of homogenized swab transport medium supernatant were serially diluted 10-fold up to 10^−10^ in phosphate buffer saline (Thermo Fisher Scientific, Illkirch, France). Then, 50 μL of each dilution were plated onto Columbia agar enriched with 5% sheep blood (Becton Dickinson, Heidelberg, Germany) and incubated aerobically for 24 hours at 37°C. Furthermore, 50 μL of each dilution plated onto Columbia agar enriched with 5% sheep blood plates were placed in a zip bag (Oxoid, Dardilly, France) supplemented with an anaerobic generator (Becton Dickinson) incubated in an anaerobic chamber (AES Chemunex, Combourg, France) at 37°C for 48 hours. In parallel, 100 μL of such supernatant were also inoculated for 1 day, 3 days, 7 days and 10 days into BACT/ALERT flasks (bioMérieux, Marcy l’Etoile, France) and YCFA liquid medium (DSMZ: Deutsche Sammlung von Mikroorganismen und Zellkulturen, Germany) (https://www.dsmz.de/microorganisms/medium/pdf/DSMZ_Medium1611.pdf), both supplemented with 2 mL of 0.2 μm-filtered rumen fluid and 2 mL of sterile defibrinated horse blood (bioMérieux). After each incubation period, 100 μL of medium supernatant were collected and processed as described above for direct inoculation and aerobic and anaerobic incubation. All visible colonies were identified by Matrix Assisted Laser Desorption Ionization—Time of Flight mass spectrometry (MALDI-TOF-MS) (Microflex, Bruker Daltonik, Bremen, Germany) as previously described [12]. Such identified *B*. *trematum* colonies were subcultured on 5% sheep blood Columbia agar at 37°C for 24 hours for further analyses, as below.

### *B*. *trematum* antimicrobial susceptibility testing

Antibiotic susceptibility testing was performed using the agar disk diffusion and E-test (bioMérieux) methods. Briefly, a 0.5-McFarland bacterial suspension in 0.85% saline was spread out using sterile cotton on Mueller Hinton agar plates (Bio-Rad), in the presence of the appropriate antibiotic disk or E-test for a series of 35 different antibiotics. After 24 hours of incubation at 37°C, inhibition zone diameters measured around the disk using SIRscan 2000 Automatic reader (i2a, Montpellier, France) were translated in minimal inhibitory concentration (MIC) values according to 2022 EUCAST (European Committee on Antimicrobial Susceptibility Testing) recommendations, using the critical concentration PK/PD, not related to a species to categorise the isolate as susceptible (S), intermediate (I) or resistant; or directly read from E-test [13]. As for controls, a comparative approach of antibiotic susceptibility included two *B*. *trematum* Archet-1 and Archet-2 clinical strains isolated in the bacteriology laboratory of the CHU in Nice, France.

### *B*. *trematum* cytotoxicity assay

Vero E6 cells (ATCC CRL-1586, Manassas, USA) cultured in 1x minimum Eagle’s medium (MEM) with 4% heat-decomplemented foetal bovine serum (Gibco Life Technologies, Paisley, UK) and 1% glutamine were dispensed into a 96-well flat-bottom opaque cell culture plate at an average of 10^5^ cells per well and incubated for 24 hours at 37°C under a 5% CO_2_ atmosphere. *B*. *trematum* strains at 1 McFarland growth were pelleted by centrifugation at 112 g for 10 minutes. The supernatant was then 0.2 μm-filtered and the pellet was washed with PBS and resuspended in cell culture medium. The 96-well plates were inoculated with either 0.2 μm filtered bacterial supernatants or the *B*. *trematum* strains at a multiplicity of infection (MOI) of 100 and then incubated for two hours at 37°C under a 5% CO_2_ atmosphere. Two types of negative controls for cytotoxicity were introduced in the experiments: (1) wells containing only cells and cell culture medium, and (2) wells supplemented with 0.2 μm-filtered bacterial culture medium. A positive control was established with digitonin (Sigma-Aldrich, St Quentin Fallavier, France). Cytotoxic activity was measured using the CellTiter-Glo kit (Promega, Charbonnières-les-Bains, France), a viable cell number count method-based quantification of ATP, an indicator of metabolically active cells (https://www.promega.com).

### *B*. *trematum* whole genome sequencing

Total genomic DNA was extracted from each of the four *B*. *trematum* isolates using the EZ1 protocol according to the manufacturer’s instructions (Qiagen, Hilden, Germany). Briefly, 200 μL of *B*. *tremarum* suspension mixed with 200 μL of G2 buffer and 20 μL of proteinase K (Qiagen) was incubated at 56°C for three hours. DNA extracted from 200 μL of mixture and eluted in a 50-μL volume was qualitatively and quantitatively evaluated using Qubit dsDNA High Sensitivity Assay Kit (Life Technology, Villebon-sur-Yvette, France). Genome sequencing was conducted combining the Oxford Nanopore GridION (Oxford Nanopore Technologies, Oxford, UK) and Illumina Miseq (Illumina, San Diego, CA, USA) sequencing platforms. Illumina and Nanopore sequencing reads were quality-controlled using FastQC (v0.11.5) software (Babraham Bioinformatics—FastQC A Quality Control tool for High Throughput Sequence Data)and NanoPlot (version 1.19.0) [14], respectively. Adapter sequence and low-quality bases were removed using Trimmomatic (v0.39) [15] for Illumina data and porechop (v0.2.4) [16] for Nanopore reads and sequences < 1,000 bases for Nanopore reads were removed using filtlong (v0.2.1) (settings, min = 1000, K = 90) [17]. Clean Nanopore and Illumina reads were combined by a hybrid assembly approach using Unicycler (v0.5.0) [18]. *De novo* assembly graphs were visualised using Bandage v0.8.1 [19]. Contigs were combined into a single molecule using the online Fasta dataset joiner (http://usersbirc.au.dk/biopv/php/fabox/fasta_joiner.php) [20].

### Genome annotation and comparative genomics

The quality and contiguity of assembled genomes was assessed using QUAST (5.0.2) [21] and the completeness of the assembled genome was assessed using Busco (v5.1.1) [22]. Single-copy orthologous gene sets were searched, and gene content was compared with 569 genes in the *Betaproteobacteria* database (available at BUSCO v1 (ezlab.org)). Final genome assembly adjustments were performed by polishing using Pilon (v1.24) [23] with Illumina’s own reads and the quality was then evaluated. Protein coding sequences (CDSs), tRNAs and rRNAs were predicted using Prokka [24] and the number of pseudogenes was calculated using the script get_pseudo.pl (VFDB: Virulence Factor Database (mgc.ac.cn)). Pathogenicity and antibiotic resistance analyses were conducted using the VFDB (Virulence Factors of Pathogenic Bacteria) [25] and CARD (Comprehensive Antibiotic Research Database) (The Comprehensive Antibiotic Resistance Database (mcmaster.ca)) [26].

## Results

### Patients

The study included nine patients with cutaneous chronic ulcers, seven of whom were males, with a mean age of 44.11 years (ranging from 14 to 83 years). All ulcers were primarily the lower extremities (7/9), and ulcers had been present for one month or more. Three patients had ulcers for more than one year and two were associated with the recurrence of lesions and diagnosed as Buruli ulcers more than ten years earlier.

### *M*. *ulcerans* DNA detection

A total of 18 swabs (nine lesion swabs and nine lateral healthy skin control swabs) were tested for *M*. *ulcerans* in the presence of negative controls included in each PCR run remained negative for all targeted sequences, insertion sequences IS2404 and IS2606 and the ketoreductase-B (KR-B) gene targeted for identification of *M*. *ulcerans*. IS2404 was detected in all lesion swabs, KR-B was detected in seven and IS2606 in three of them. However, IS2404 and KR-B were detected in five control swabs and IS2606 in two control swabs. According to the previously defined criteria, 12 (66.66%) were positive for *M*. *ulcerans* including seven with positive lesions and five for lateral controls (Table 2). Four of seven patients with *M*. *ulcerans*-positive chronic ulcer also harboured *M*. *ulcerans* in the auto control swab.

**Table 2:** Buruli ulcer PCR test results. Sample are considered positive for *M*. *ulcerans* if the KR-B gene was detected with a Ct < 40 cycles and at least one of the two insertion sequence PCRs resulted in a Ct < 40 cycles and KR-B was detected with a Ct < 40 cycles.

Patient’s code	Lesion				Control			
	IS2404	IS2606	KrB	PCR Interpretation	IS2404	IS2606	KrB	PCR Interpretation
	(Ct)	(Ct)	(Ct)		(Ct)	(Ct)	(Ct)	
**CI016**	**+(35)**	**-**	**+(34)**	**Positive**	**+(35)**	**+(31)**	**+(35)**	**Positive**
**CI014**	**+(36)**	**+(36)**	**+(34)**	**Positive**	**-**	**-**	**-**	**Negative**
**CI027**	**+(34)**	**-**	**+(35)**	**Positive**	**+(31)**	**+(28)**	**+(32)**	**Positive**
**CI032**	**+(36)**	**-**	**-**	**Negative**	**-**	**-**	**-**	**Negative**
**CI040**	**+(34)**	**-**	**+(36)**	**Positive**	**+(33)**	**-**	**+(34)**	**Positive**
**CI053**	**+(37)**	**-**	**-**	**Negative**	**+(32)**	**-**	**+(32)**	**Positive**
**CI062**	**+(31)**	**+(33)**	**+(34)**	**Positive**	**+(33)**	**-**	**+(33)**	**Positive**
**CI070**	**+(30)**	**+(34)**	**+(32)**	**Positive**	**-**	**-**	**-**	**Negative**
**CI083**	**+(28)**	**-**	**+(29)**	**Positive**	**-**	**-**	**-**	**Negative**

### Swab culturomics

**Fig 1:**
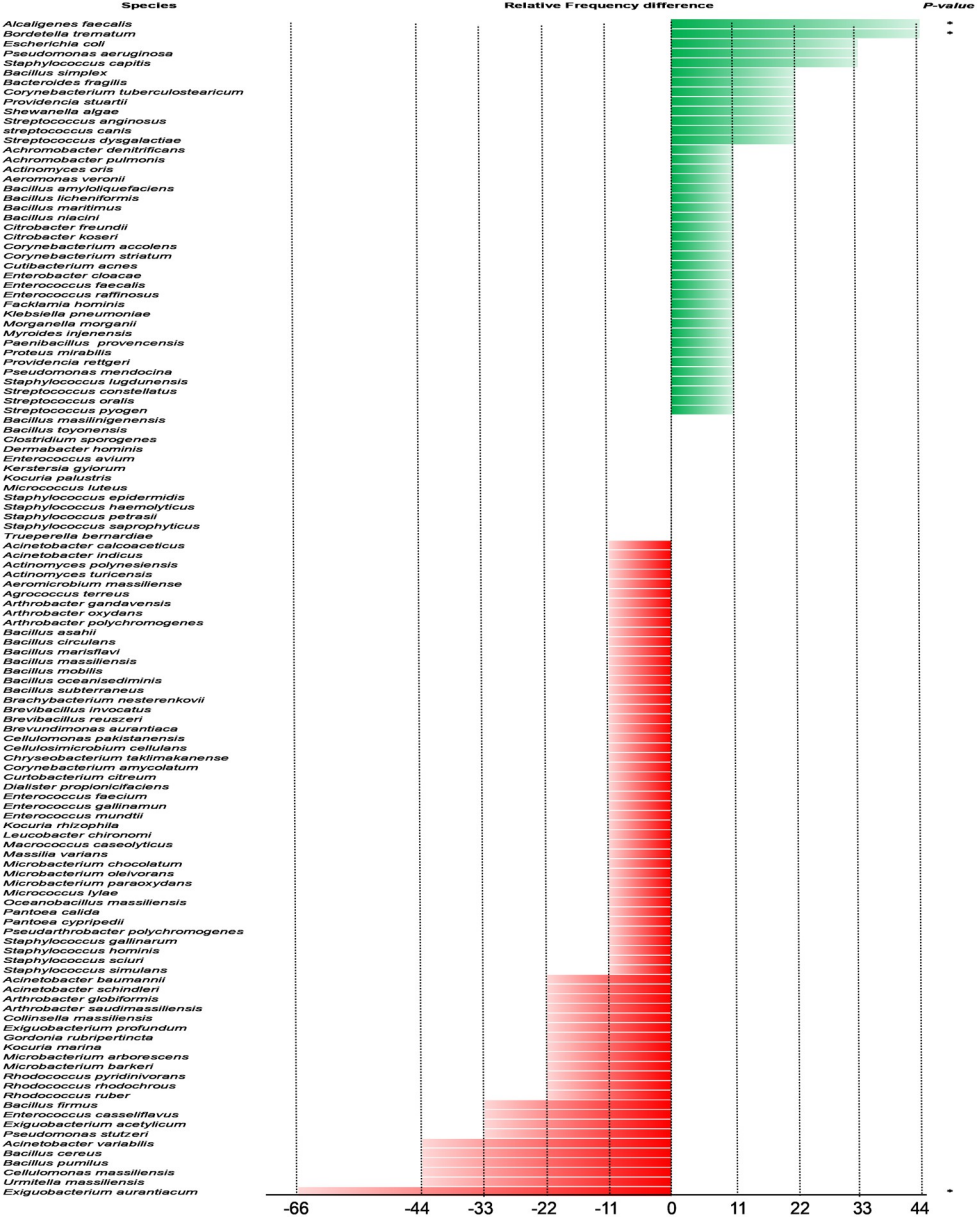
Bacterial species frequency difference isolated from 18 samples consisting of 9 ulcer samples (green bar) and 9 self-control samples of healthy skin (red bar). Frequency is expressed as a percentage (%).

Analysis of the microbiological composition of the swabs taken from the chronic ulcer samples of the nine patients revealed 99 bacterial isolates belonging to 61 different species, of which 21 (34.43%) were gram-negative and 40 (65.57%) were gram-positive and classified into four phyla: *Firmicutes* (49.18%), *Proteobacteria* (31.15%), *Actinobacteria* (16.39%) and *Bacteroidetes* (3.28%). Twenty-five species (44.64%) were grown from at least two samples, while thirty-six (55.36%) species were grown from a single sample. Staphylococci, *Bacillus* and streptococci were the most commonly isolated groups. In terms of the species isolated and the patients, six species (*Alcaligenes faecalis* (*A*. *faecalis*), *Bordetella trematum* (*B*. *trematum*), *Escherichia coli*, *Micrococcus luteus*, *Staphylococcus epidermidis* and *Staphylococcus capitis*) were the most frequently isolated in four of the nine patients (Fig 1). One hundred and forty-eight bacterial isolates representing 90 different bacterial species, sorted into *Firmicutes* (41.11%), *Actinobacteria* (40.00%), *Proteobacteria* (17.78%) and *Bacteroidetes* (1.11%), were identified in the auto-control skin samples. Seventy-three (80.22%) of the species were gram positive and seventeen (18.62%) were gram negative, divided into 42 genera. The genus *Bacillus* was the most isolated, followed by *Staphylococcus* and *Enterococcus*. The most common species in the samples were *Bacillus cereus* and *Exiguobacterium aurantiacum* (*E*. *aurantiacum*), found in six of the nine swabs (Fig 1).

### Comparative microbiology

**Fig 2:**
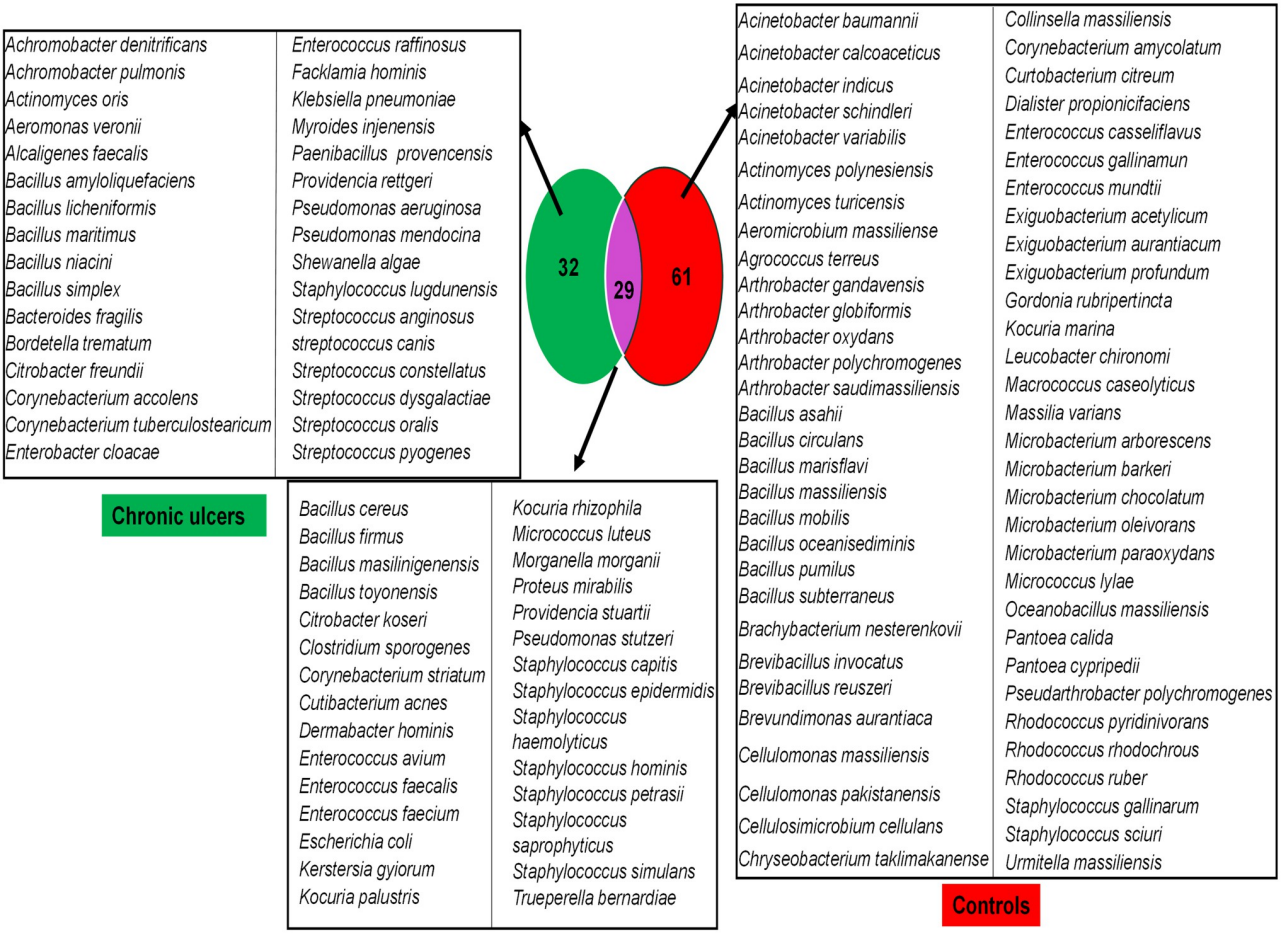
Venn diagram representing species isolated from chronic cutaneous tropical ulcers and control samples, Côte d’Ivoire.

A total of 122 different bacterial species were isolated and identified from chronic ulcer and control swabs. Thirty-two species were specifically isolated from chronic ulcer swabs, 61 from control swabs and 29 species were common to both types of swabs (Fig 2).

**Fig 3:**
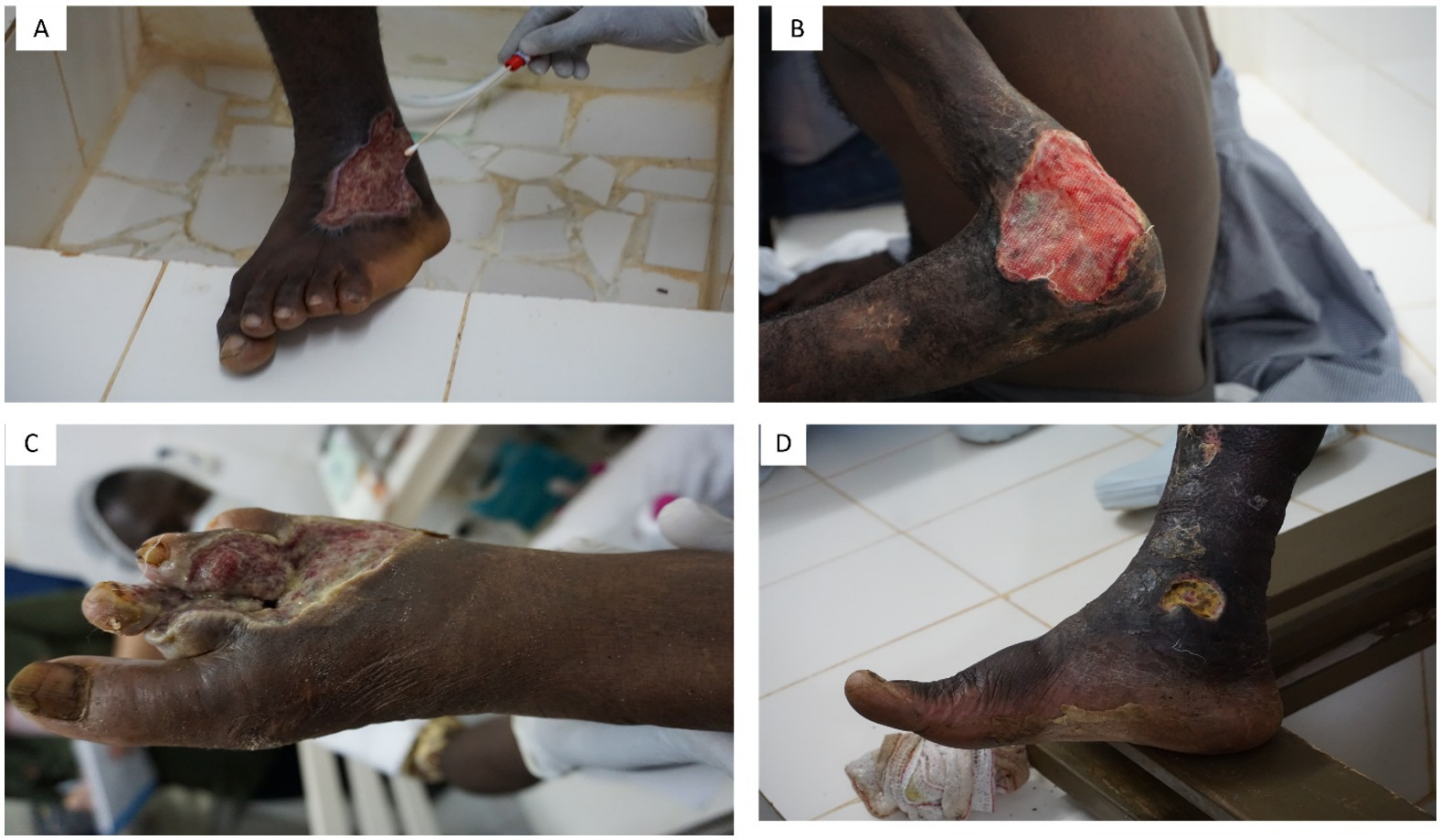
**Clinical presentation of *B*. *trematum* culture-positive ulcers**: (A) Patient CI016, a 16-year-old patient from whom *B*. *trematum* strain CI016 was isolated. (B) Patient CI040, 40-year-old patient from whom *B*. *trematum* CI040 was isolated. (C) Patient CI070, 70-year-old patient from whom *B*. *trematum* CI070 was isolated. (D) Patient CI083, an 83-year-old patient from whom *B*. *trematum* CI083 was isolated. (Photography credit: M. Drancourt.)

A comparison of the bacterial repertoire between the two types of swabs revealed that *B*. *trematum* and *A*. *faecalis* were significantly associated with chronic ulcer swabs (*p* value = .025, Fisher’s exact test) and *E*. *aurantiacum* (*p* value = .04, Fisher’s exact test) was significantly associated with control swabs. *B*. *trematum* and *A*. *faecalis* microorganisms were isolated from four of the seven documented *M*. *ulcerans* Buruli ulcer swabs and not from the healthy skin control swab. In particular, the *B*. *trematum* strain CI016 was isolated from a left ankle swab from a 16-year-old male, along with 13 other bacterial species (Fig 3A, Table 3). *B*. *trematum* strain CI 040 was isolated from swab from a reopened scar on the left elbow of a 40-year-old man, along with five other bacterial species (Fig 3B, Table 3). *B*. *trematum* strain CI070 was co-isolated with 24 bacterial species from a swab of a right foot in a 70-year-old man (Fig 3C, Table 3). *B*. *trematum* strain CI083 was isolated from a swab of the right foot of an 83-year-old man, along with eight other bacterial species (Fig 3D, Table 3).

**Table 3:** Bacterial species isolated from ulcer swabs (and not from healthy skin swab) positive for *M*. *ulcerans* (PCR detection) and for *B*. *trematum* (culture detection), in Côte d’Ivoire. Patients coded as CI0X.

CI016	CI040	CI070	CI083
*Alcaligenes faecalis*	*Alcaligenes faecalis*	*Alcaligenes faecalis*	*Achromobacter denitrificans*
*Bacillus firmus*	*Bacteroides fragilis*	*Bacillus cereus*	*Achromobacter pulmonis*
*Bacillus maritimus*	*Bordetella trematum*	*Bacteroides fragilis*	*Bordetella trematum*
*Bacillus masilinigenensis*	*Pseudomonas aeruginosa*	*Bordetella trematum*	*Citrobacter koseri*
*Bacillus niacini*	*Staphylococcus epidermidis*	*Citrobacter koseri*	*Enterococcus faecalis*
*Bordetella trematum*	*Staphylococcus saprophyticus*	*Clostridium sporogenes*	*Escherichia coli*
*Corynebacterium striatum*		*Corynebacterium striatum*	*Klebsiella pneumoniae*
*Cutibacterium acnes*		*Corynebacterium tuberculostearicum*	*Proteus mirabilis*
*Escherichia coli*		*Enterococcus avium*	*Providencia stuartii*
*Morganella morganii*		*Enterococcus raffinosus*	*Pseudomonas aeruginosa*
*Pseudomonas aeruginosa*		*Escherichia coli*	*Staphylococcus capitis*
*Staphylococcus capitis*		*Facklamia hominis*	*Streptococcus oralis*
*Staphylococcus haemolyticus*		*Kerstersia gyiorum*	
*Staphylococcus lugdunensis*		*Kocuria palustris*	
*Streptococcus dysgalactiae*		*Myroides injenensis*	
		*Proteus mirabilis*	
		*Providencia rettgeri*	
		*Providencia stuartii*	
		*Shewanella algae*	
		*Staphylococcus capitis*	
		*Streptococcus anginosus*	
		*Streptococcus canis*	
		*Streptococcus constellatus*	
		*Streptococcus pyogenes*	
		*Trueperella bernardiae*	

### *B*. *trematum* antimicrobial susceptibility testing

Six *B*. *trematum* isolates, including four isolates recovered in this study and two isolates previously isolated at the Nice hospital laboratory (strains Archet 1 and 2), yielded a similar pattern of *in vitro* susceptibility and resistance to 35 antibiotics (Table 4). As for beta-lactamines, isolates were susceptible to amoxicillin (MIC, 1–2 mg/L), amoxicillin/clavulanic acid (MIC, 0.5–1 mg/L), piperacillin (MIC, 1–1.5 mg/L) and piperacillin/tazobactam (MIC, 0.75 mg/L), imipenem (MIC, 0.38 mg/L), ertapermen (MIC, 0.012–0.016 mg/L) and meropenem (MIC, 0.064–0.125 mg/L). However, they were resistant to mecilliman and aztreonam (MIC>256 mg/L). Further, the six isolates were susceptible to cefepime, ceftazidime and ceftazidime/avibactam with MICs of 3 mg/L to 6 mg/L and were resistant to cefotaxime (MIC > 32 mg/L), cefoxitin (MIC > 256 mg/L) and ceftolozane-tazobactam (MIC between 12 mg/L and 16 mg/L). As for aminoglycosides, the isolates were resistant to gentamicin (MIC range 6 mg/L to 8 mg/L), tobramycin (MIC range 3 mg/L to 6 mg/L), amikacin (MIC range 12 mg/L to 32 mg/L) and streptomycin (MIC, 256 mg/L). As for fluoroquinolones, all isolates were resistant to ciprofloxacin (MIC range 6 mg/L to >32 mg/L) and ofloxacin (MIC range 3–16 mg/L), whereas *B*. *trematum* strains CI040, CI083 and Archet 2 showed resistance to levofloxacin (MIC range 1–2 mg/L) while strains CI016, CI070 and Archet 1 were susceptible with MIC range 0.5–0.75 mg/L. Resistance was observed for rifampicin (MIC, >32 mg/L) and fosfomycin (MIC, 256 mg/L to >1024 mg/L). As for clarithromycin, the MIC = 16 mg/L, not interpretable using EUCAST standards, was here interpreted as resistant. The highest susceptibility MIC accepted by EUCAST is 8 mg/L (for mycobacteria). MIC values for colimycine varied from 0.25 mg/L to 1 mg/L. In addition, *B*. *trematum* strain-CI016 showed resistance to trimethoprim-sulfamethoxazole (MIC, 32 mg/L) contrary to the five other isolates.

**Table 4:** Antimicrobial susceptibility testing (ATS) results for four *B*. *trematum* isolates from chronic cutaneous ulcers, Côte d’Ivoire.

		Strain CI016		Strain CI040		Strain CI070		Strain CI083		Strain Archet 1		Strain Archet 2	
Antibiotic family	Antimicrobial agent	Inhibition diameter (mm)	MIC (mg/L)	Inhibition diameter (mm)	MIC (mg/L)	Inhibition diameter (mm)	MIC (mg/L)	Inhibition diameter (mm)	MIC (mg/L)	Inhibition diameter (mm)	MIC (mg/L)	Inhibition diameter (mm)	MIC (mg/L)
Penicillins	Amoxicillin	31	1,5	31	2	33	1	34	1,5	33	1,5	30	1
	Amoxicillin/clavulanic acid	30	0,75	30	1	31	0,75	29	0,75	28	1	29	0,5
	Ticarcillin	35	-	36	-	38	-	37	-	36	-	34	-
	Ticarcillin /Clavulanic acid	36	-	35	-	37	-	35	-	33	-	35	-
	Mecillinam	6	>256	6	>256	6	>256	6	>256	16	>256	14	>256
	Piperacillin	28	1	32	1,5	29	1	30	1	28	1	30	1
	Piperacillin/ Tazobactam	31	0,75	31	0,75	32	0,75	33	0,5	32	0,75	29	0,75
	Temocillin	6	-	6	-	6	-	6	-	12	-	10	-
Cephalosporins	Cephalexin	16	-	15	-	17	-	14	-	16	-	17	-
	Cefoxitin	6	>256	6	>256	6	>256	6	>256	6	>256	6	>256
	Cefepime	25	6	28	6	27	4	26	3	22	4	26	4
	Ceftazidime	21	3	21	3	22	2	22	2	23	3	21	2
	Cefotaxime	11	>32	6	>32	16	>32	6	>32	14	>32	14	>32
	Ceftolozane + tazobactam	15	16	17	16	20	12	16	12	6	12	6	12
	Cefixime	6	16	6	24	6	12	6	16	6	16	12	16
	Ceftazidime/avibactam	22	4	20	4	21	3	20	2	6	4	26	3
Carbapenems	Imipenem	34	0,38	36	0,38	34	0,38	37	0,38	35	0,38	34	0,38
	Meropenem	36	-	40		37	-	40	-	36	-	33	-
	Ertapenem	36	0,012	35	0,012	35	0,008	40	0,012	35	0,012	36	0,016
Monobactams	Aztreonam	6	>256	11	>256	13	96	9	>256	13	>256	14	>256
Aminoglycosides	Gentamicin	11	8	10	6	13	8	11	8	19	8	23	6
	Amikacin	17	24	17	32	18	24	17	12	24	24	26	32
	Streptomycin	-	256	-	256	-	256	-	256	-	256	-	256
	Tobramycin	12	6	10	6	11	4	10	3	29	6	29	6
Tetracyclines	Tigecycline	32	-	32	-	31	-	25	-	6	-	17	-
Fluoroquinolones	Nalidixic acid	23	-	25	-	21	-	21	-	13	-	24	-
	Ciprofloxacin	17	6	11	>32	16	3	11	12	6	>32	6	6
	Levofloxacin	20	0,75	17	1	20	0,75	15	1,5	12	0,5	11	2
	Ofloxacin	19	3	16	4	19	3	11	6	6	2	6	16
Sulfonamides	Trimethoprim Sulfamethoxazole	6	32	16	0,25	18	25	16	0,25	16	0,38	18	0,25
Macrolide	clarithromycin	-	16	-	16	-	16	-	16	-	16	-	16
Polymicin	Colistin	24	0,25	24	0,25	23	0,25	23	0,25	20	0,25	19	1
Nitrofural	Furans	8	-	6	-	6	-	6	-	18	-	16	-
Others antibiotics	Fosfomycine	21	256	21	>1024	13	>1024	21	>1024	29	256	18	>1024
	Rifampicine	-	>32	-	>32	-	>32	-	>32	-	>32	-	>32

STA was performed by electronic and disk diffusion test methods and MIC results are given in mm and mg / L. respectively. Colour code: green = susceptibility, red: Resistant, yellow: intermediate and white: No EUCAST interpretation rule.

### Cytotoxicity assay

**Fig 4:**
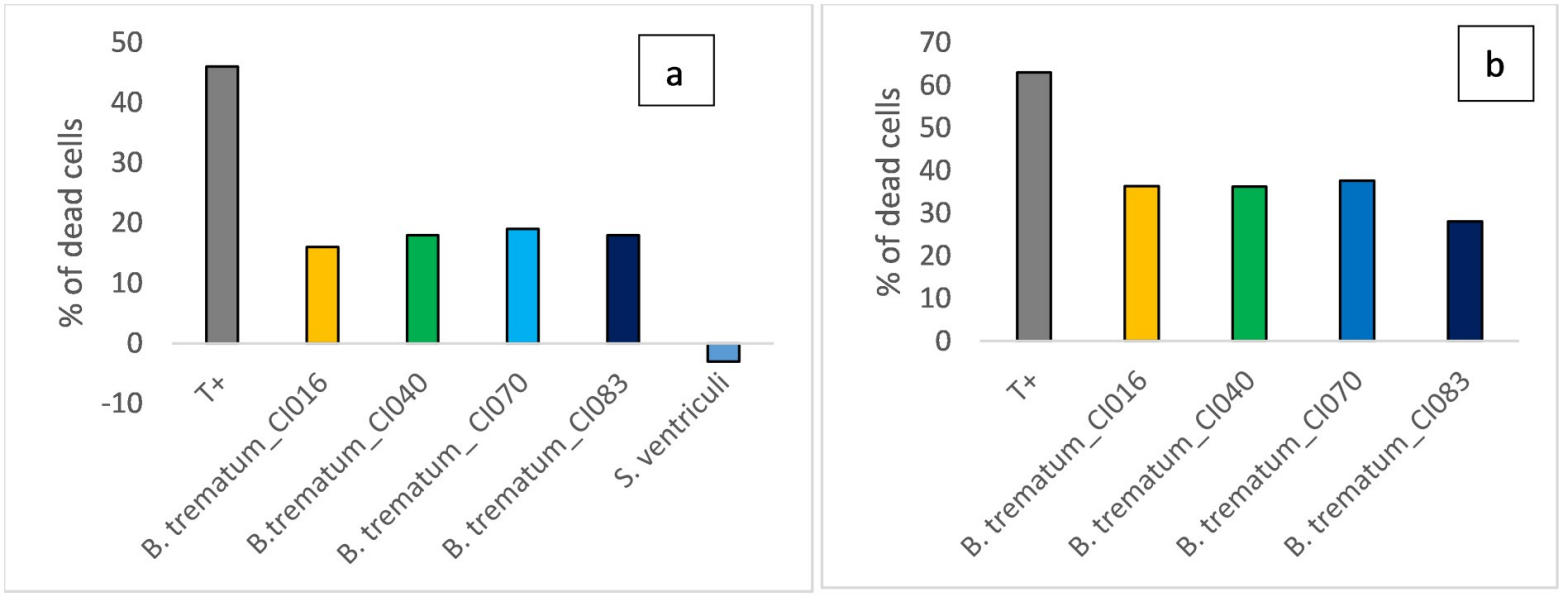
*trematum* cytotoxicity for Vero E6 cells. ***B*.** (a): Confluent Vero E6 cells were incubated with a bacterial suspension (MOI = 100). After two hours of incubation, the CellTiter-Glo kit was used for cytotoxicity testing. For the negative control of cell death, cells were incubated only with 1x minimum Eagle’s medium (MEM) with 4% heat-decomplemented foetal calf serum and 1% glutamine. Then a *Sarcina ventriculi* known to be devoid of cytotoxic activity was used as a control of negative cytotoxic activity. Digitonin was used as a positive control for cytotoxic activity. (b): Confluent Vero E6 cells were incubated with filtered and lyophilised bacterial supernatant resuspended in 1x minimum Eagle’s medium (MEM) with 4% heat-decomplemented foetal calf serum and 1% glutamine. After two hours of incubation, the CellTiter-Glo kit was used for cytotoxicity testing. For the negative control of cell death, cells were incubated only with 1x minimum Eagle’s medium (MEM) with 4% heat-decomplemented foetal calf serum and 1% glutamine. The TCSB medium used for bacterial culture, treated under the same conditions, was used to control the cytotoxic activity of the medium. This activity was then deduced from the activity of B. trematum isolates. Digitonime was used as a positive control for cytotoxic activity.

Testing the *B*. *trematum* cytotoxic effect on Vero E6 cells indicated cytotoxic activity of *B*. *trematum* isolates on Vero E6 cells. The activity was almost similar for all isolates but with the supernatant a slightly higher cytotoxic activity was observed (Fig 4).

### Genomic analyses

**Fig 5:**
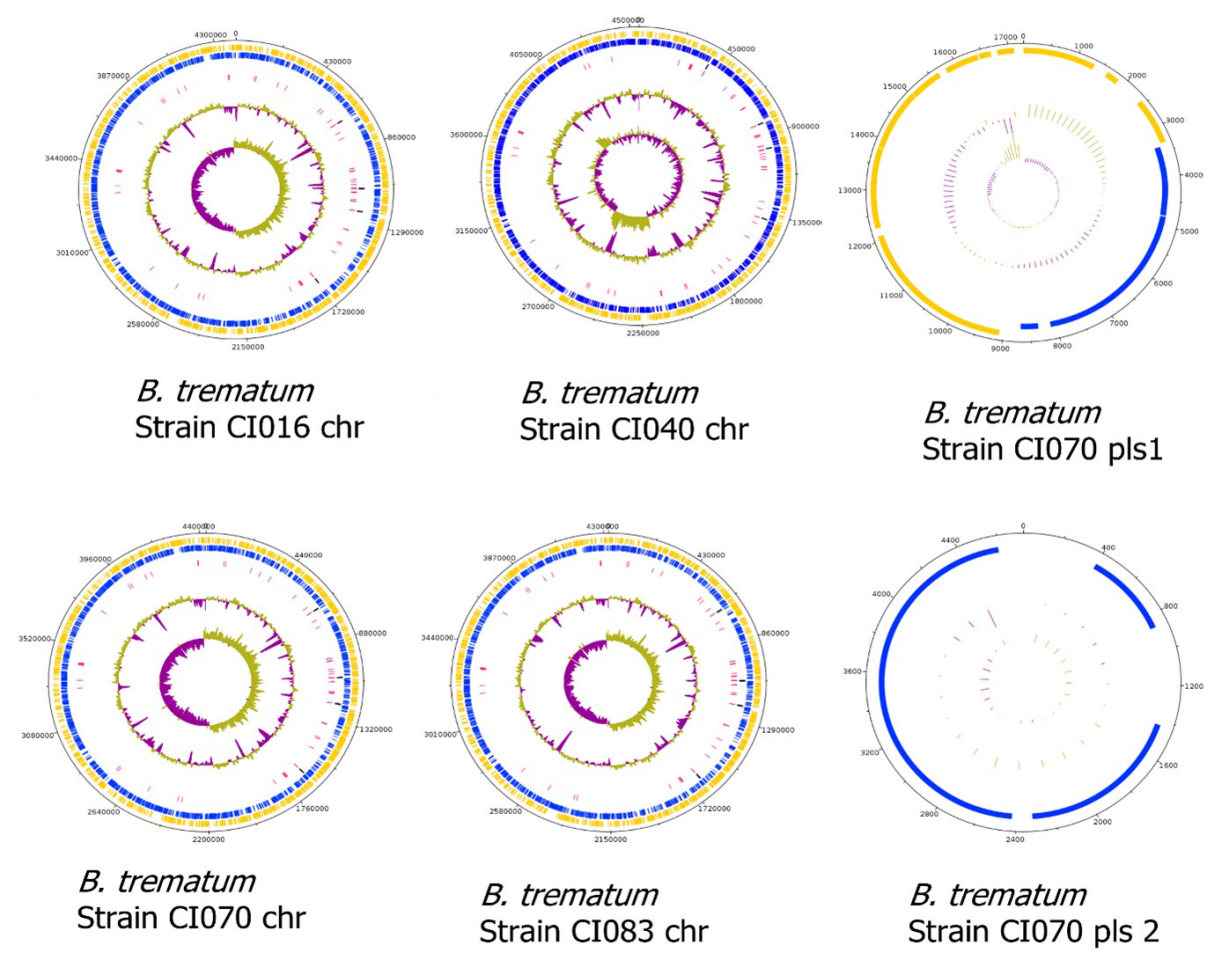
Circular genome map of *B*. *trematum*, constructed using Artemis. From outer circle to inner, genomic features were presented as: forward strand CDS (yellow), reverse strand CDS (blue), rRNA genes (black) tRNA genes (purple), GC content and GC skew.

The four investigated *B*. *trematum* WGSs produced between one and six contigs with > 96% completeness, establishing the reliability of the datasets for downstream analyses (Fig A and Table A in S1 Text). The 4 397 893-bp *B*. *trematum* CI016 strain WGS featured a single circular chromosome with 95% coverage and 99% identity to reference *B*. *trematum* strain F581 (GenBank CP016340.1); comprising 3964 coding protein sequences (CDS), 12 rRNAs, one tmRNA, 67 tRNA, 21 pseudo genes and 15 insertion sequences (IS). The 4 342 488-bp *B*. *trematum* CI070 strain WGS featured a single circular chromosome with 96% coverage and 99% identity to reference *B*. *trematum* isolate E202 (GenBank: CP049957.1) and two plasmids. The 17 274-bp plasmid I exhibited 82% coverage and 91% identity to *Citrobacter sp*. RHBSTW-00848sp plasmid (GenBank CP055914.1) and a 4729-bp plasmid II exhibited 57% coverage and 82% identity with *Edwardsiella tarda* strain 9.2 plasmid p9.2 (GenBank MG228256.1). Full chromosomal sequence annotation found 3892 CDS, 12 rRNA, one tmRNA, 67 tRNA, 17 pseudogenes and 13 IS, plasmid I has 20 CDS and plasmid II three CDS. The 4 541 719-bp *B*. *trematum* CI040 strain WGS consisted of one chromosome exhibiting 92% coverage and 97% identity with reference *B*. *trematum* strain E202 (GenBank: CP049957.1). Full genome annotation revealed 4121 CDS, 12 rRNA, 67 tRNA, one tmRNA, 21 pseudogenes and 16 IS. Finally, the 4 427 308-bp *B*. *trematum* CI083 strain genome consisted of one chromosome exhibiting 94% coverage and 97% identity with the reference *B*. *trematum* F581 strain genome sequence (GenBank CP016340.1). Its full genome sequence annotation found 3992 CDS, 12 rRNA, one tmRNA, 68 tRNA, 17 pseudogenes and 13 IS (Fig 5).

**Fig 6:**
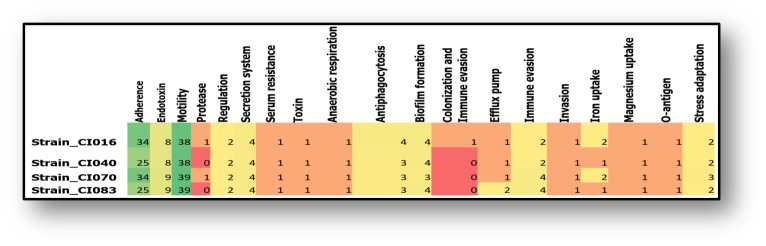
Virulence factor profiles in four *B*. *trematum* isolates from documented *M*. *ulcerans* Buruli ulcers, Côte d’Ivoire.

A total of 111, 98, 109 and 99 potential virulence factors were annotated in the genome of *B*. *trematum* strain-CI016, *B*. *trematum* strain-CI040, *B*. *trematum* strain-CI070 and *B*. *trematum* strain-CI083, respectively (Fig 6).

Specifically, a flagellar system associated with mobility in all genomes examined accounted for 34% to 39% of annotated virulence factors; in addition to pili/fimbriae-associated proteins, filamentous hemagglutinin and LPS O-antigen, involved in *Bordetella* adhesion, accounting for 25% to 31% of genes. All *B*. *trematum* isolates expressed eight genes (*pag*P, *bpl*A, *bpl*B, *bpl*C, *bpl*D, *bpl*E, *bpl*L and kdsA) encoding lipopolysaccharide biosynthesis. We also noted the presence of a gene encoding the cytolethal distending toxin B subunit (CdtB). All isolates contained genes involved in biofilm formation (*ade*F, *ade*G and *ade*H), antiphagocytic activity (*alg*W, *wcb*T), efflux (*mex*A, *mtr*D), and immune evasion, invasion, iron and magnesium absorption and stress adaptation. Strains CI016 and CI070 had protease-related genes. A search for drug resistance genes revealed potential genes encoding resistance to fluoroquinolone, tetracycline, disinfectants and antiseptics in all strains and strain CI016 had a gene *Sul*2 encoding sulphonamide resistance.

## Discussion

The first cases of Buruli ulcer were reported by Sir Albert Cook in his notes at Mengo Hospital in Kampala, Uganda in 1896 [27]. However, this tropical infection was formally described by Australian colleagues in the early 1950s, uniquely combining epidemiology, clinical and pathology features, and isolation through the culture of the acknowledged aetiological agent, *M*. *ulcerans* [28]. The then routine usage of convenient PCR (instead of fastidious culture) for the diagnosis of Buruli ulcer slightly complicated the situation after it was reported the detection of *M*. *ulcerans* DNA on apparently healthy skin, emphasizing that a positive PCR detection of this opportunistic pathogen was interpretable only on skin lesions, chiefly chronic ulcers [11]. In this study, we adopted RT-PCR diagnostic criteria more stringent that the WHO-recommended IS2404 insertion sequence positivity, adding positivity of IS2606 and KR-B gene, to confirm cases of Buruli ulcer. Stringoury was chosen to increase Buruli ulcer diagnostic specificity, with the risk to miss true cases of Buruli ulcer: two samples (CI032 and CI053) were declared negative using our criteria despite Ct values of 36 and 37 for the IS2404 sequence. These results could either indicate a low bacterial load or suggest that our criteria excluded borderline cases. Examination of the clinical context adds a further layer of complexity: patient CI032 presented a very old lesion, a reopening of the scar of an old confirmed Buruli ulcer, which could justify a low bacterial load; patient CI053 was in the early stages of the disease, which would also explain the low bacterial load. Further microbiological investigations identified a total of 25 different bacteria in cases of Buruli ulcer confirmed by PCR including *S*. *aureus* and *P*. *aeruginosa*, which have been convincingly associated with delayed healing of Buruli ulcer [5,7,29] (Table 5).

**Table 5:** The different bacterial species isolated in documented Buruli ulcer lesions. These data come from studies conducted by Yeboah-Manu *et al*., [5], Barogui *et al*., [7] and Anyim et al. [29].

Specises associated with Buruli ulcer (literature review)	Species isolated in addition in the present work
*Aeromonas hydrophila*	*Achromobacter denitrificans*
*Acinetobacter sp*	*Achromobacter pulmonis*
*Alcaligenes faecalis*	*Actinomyces oris*
*Burkholderia cepacia*	*Bacillus amyloliquefaciens*
*Chrysemonas luteola*	*Bacillus cereus*
*Citrobacter koseri*	*Bacillus firmus*
*Coagulase negative Staphylococci*	*Bacillus maritimus*
*Enterobacter cloacae*	*Bacillus masilinigenensis*
*Enterobacter sakazalii*	*Bacillus niacini*
*Enterococcus gallinum*	*Bacillus simplex*
*Escherichia coli*	*Bacillus toyonensis*
*Flavibaterium oryzihabitans*	*Bacteroides fragilis*
*Group A stretococci*	*Bordetella trematum*
*Klebsialla pneumoniae*	*Citrobacter freundii*
*Kluyvera ascorbata*	*Clostridium sporogenes*
*Moraxella spp*	*Corynebacterium accolens*
*Morganella morganii*	*Corynebacterium striatum*
*Pasteuralla multocida*	*Corynebacterium tuberculostearicum*
*Proteus miriabilis*	*Cutibacterium acnes*
*Proteus vulgaris*	*Dermabacter hominis*
*Providencia rettgeri*	*Enterococcus avium*
*Providencia stuartii*	*Enterococcus faecalis*
*Pseudomonas aeruginosa*	*Enterococcus faecium*
*Pseudomonas pseudomallei*	*Enterococcus raffinosus*
*Staphylococcus aureus*	*Facklamia hominis*
*Vibrio alginolyticus*	*Kerstersia gyiorum*
	*Klebsiella pneumoniae*
	*Kocuria palustris*
	*Kocuria rhizophila*
	*Micrococcus luteus*
	*Myroides injenensis*
	*Paenibacillus provencensis*
	*Pseudomonas mendocina*
	*Pseudomonas stutzeri*
	*Shewanella algae*
	*Streptococcus anginosus*
	*Streptococcus canis*
	*Streptococcus constellatus*
	*Streptococcus oralis*
	*Trueperella bernardiae*

Using culturomics and auto control as previously reported in investigation of diabetic ulcers (as an example) [30], our study significantly expanded the bacterial repertory of Buruli ulcers confirmed by PCR, adding 40 bacterial species to the previous reports, revealing a previously unreported prevalence of *B*. *trematum* co-infection with *M*. *ulcerans* in this situation of tropical chronic cutaneous ulcers. *B*. *trematum* has been previously delineated as a unique bacterial species after a polyphasic investigation of ten isolates, including six isolates recovered from open wounds in patients, most of whom were European in origin [31]. *B*. *trematum* was further isolated in thirteen patients presenting with chronic cutaneous ulcers sometimes extending to underlying tissues and bones, and most patients originated from European countries [32–37], the United States [38], Brazil [39], Argentina [40] and Japan [41]. However, *B*. *trematum* has never been previously reported as a co-pathogen with *M*. *ulcerans*.

Genomic analysis revealed virulence factors associated with colonisation (adhesin), biofilm formation and endotoxin production; all factors which may contribute to delayed Buruli ulcer healing, as previously reported for *S*. *aureus* or *P*. *aeruginosa* [5,6]. Moreover, the four *B*. *trematum* isolates here recovered from *M*. *ulcerans*-positive chronic ulcers were all producing cytotoxin. Indeed, *B*. *trematum* is the only *Bordetella* species which encodes for cytolethal distending toxin, a toxin that causes DNA damage and cell cycle arrest also encoded by other gram-negative pathogens such as *Haemophilus ducreyi* [42–43]. Lastly, our observations may be relevant for the medical management of Buruli ulcer patients in West Africa as the natural *in vitro* antibiotic susceptibility profile of *B*. *trematum* sharply differed from the one of *M*. *ulcerans* [44]. Here, *B*. *trematum* isolates were consistently resistant to rifampicin and clarithromycin, the WHO-endorsed antibiotic combination for treating Buruli ulcer in West Africa; while previously reported resistance to some fluoroquinolones [32,33,35] would have compromised success when treating Buruli ulcer (mixed with *B*. *trematum*) in Australia. Further, *B*. *trematum* isolates were resistant to aminoglycosides, as previously reported [35,45], whereas aminoglycosides were used until recently in West Africa to treat Buruli ulcer [46,47].

Present study offers one more example of polymicrobial chronic tropical ulcer: our study echoes previous microscopic observations of mixed spirochetes and fusobacteria in tropical ulcers in four different tropical countries (Zambia, Gambia, southern India and Papua New Guinea); recently complemented by metagenomic confirmation of *Fusobacterium necrophorum* in samples from Papua New Guinea [48,49]. Understanding microbial diversity in tropical ulcers may offer valuable insights into the diagnosis and management of Buruli ulcer, particularly in cases where co-infections may play a role.

In conclusion, we propose that a mixed *M*. *ulcerans*-*B*. *trematum* chronic tropical cutaneous infection is one form of Buruli ulcer warranting specific attention and diagnosis. In this perspective, further research incorporating a larger number of chronic tropical ulcers cases in different Buruli ulcer endemic West African countries is needed to understand the actual contribution of toxin-secreting *B*. *trematum* strains to this situation and the opportunistic role of toxin-secreting *B*. *trematum* strains as co-pathogens with *M*. *ulcerans*, taken into consideration the unique antibiotic susceptibility profile of this bacterium; in particular in patients who fail to respond to initial *M*. *ulcerans* treatment.

## Supporting information

S1 Text *Bordetella trematum* isolates genome assembly characteristics. (DOCX)

## References

[1] Toppino S, N’Krumah RTAS, Kone BV, Koffi DY, Coulibaly ID, Tobian F, et al. Skin wounds in a rural setting of Côte d’Ivoire: Population-based assessment of the burden and clinical epidemiology. PLoS Negl Trop Dis. 2022;16(10):e0010608. 36227839 10.1371/journal.pntd.0010608 PMC9560139

[2] Yotsu RR. Integrated Management of Skin NTDs-Lessons Learned from Existing Practice and Field Research. Trop Med Infect Dis. 2018;3(4):120. doi: 10.3390/tropicalmed3040120 30441754 PMC6306929

[3] Zingue D, Bouam A, Tian RBD, Drancourt M. Buruli Ulcer, a Prototype for Ecosystem-Related Infection, caused by *Mycobacterium ulcerans*. Clin Microbiol Rev. 2017;31(1):e00045–17. 29237707 10.1128/CMR.00045-17 PMC5740976

[4] Guarner J, Bartlett J, Whitney EA, Raghunathan PL, Stienstra Y, Asamoa K, et al. Histopathologic features of *Mycobacterium ulcerans* infection. Emerg Infect Dis. 2003;9(6):651–656. 12780997 10.3201/eid0906.020485 PMC3000137

[5] Yeboah-Manu D, Kpeli GS, Ruf MT, Asan-Ampah K, Quenin-Fosu K, Owusu-Mireku E, et al. Secondary bacterial infections of Buruli ulcer lesions before and after chemotherapy with streptomycin and rifampicin. PLoS Negl Trop Dis. 2013;7(5):e2191. doi: 10.1371/journal.pntd.0002191 23658847 PMC3642065

[6] Van Leuvenhaege C, Vandelannoote K, Affolabi D, Portaels F, Sopoh G, de Jong BC, et al. Bacterial diversity in Buruli ulcer skin lesions: Challenges in the clinical microbiome analysis of a skin disease. PLoS One. 2017;12(7):e0181994. doi: 10.1371/journal.pone.0181994 28750103 PMC5531519

[7] Barogui YT, Klis S, Bankolé HS, Sopoh GE, Mamo S, Baba-Moussa L, et al. Towards rational use of antibiotics for suspected secondary infections in Buruli ulcer patients. PLoS Negl Trop Dis. 2013;7(1):e2010. doi: 10.1371/journal.pntd.0002010 23359827 PMC3554522

[8] Hammoudi N, Dizoe S, Saad J, Ehouman E, Davoust B, Drancourt M, et al. Tracing *Mycobacterium ulcerans* along an alimentary chain in Côte d’Ivoire: A one health perspective. PLoS Negl Trop Dis. 2020;14(5):e0008228. 32463813 10.1371/journal.pntd.0008228 PMC7255608

[9] Coulibaly B, Dibi KP, Diobo KS, Koli BZ. ‘Répercussions socio-économiques de l’ulcère de Buruli en Côte d’ivoire. Exemple de la région du bélier et du district autonome de Yamoussoukro’. Revue de Géographie Tropicale et d’Environnement. 2015;2(1):16–25.

[10] Fyfe JA, Lavender CJ, Johnson PD, Globan M, Sievers A, Azuolas J, et al. Development and application of two multiplex real-time PCR assays for the detection of *Mycobacterium ulcerans* in clinical and environmental samples. Appl Environ Microbiol. 2007;73(15):4733–40. 17526786 10.1128/AEM.02971-06 PMC1951036

[11] Hammoudi N, Cassagne C, Million M, Ranque S, Kabore O, Drancourt M, et al. Investigation of skin microbiota reveals *Mycobacterium ulcerans-Aspergillus* sp. trans-kingdom communication. Sci Rep. 2021 Feb 12;11(1):3777. 33580189 10.1038/s41598-021-83236-7 PMC7881091

[12] Naud S, Khelaifia S, Mbogning Fonkou MD, Dione N, Lagier JC, Raoult D. Proof of Concept of Culturomics Use of Time of Care. Front Cell Infect Microbiol. 2020; 10:524769. doi: 10.3389/fcimb.2020.524769 33330116 PMC7719802

[13] Société Française de Microbiologie (SFM), EUCAST. Comité de l’antibiogramme de la Société Française de Microbiologie: recommendations 2022, V.1.0 May. 2022. https://www.sfm-microbiologie.org/wp-content/uploads/2022/05/CASFM2022_V1.0.pdf.

[14] De Coster W, D’Hert S, Schultz DT, Cruts M, Van Broeckhoven C. NanoPack: visualizing and processing long-read sequencing data. Bioinformatics. 2018;34(15):2666–2669. doi: 10.1093/bioinformatics/bty149 29547981 PMC6061794

[15] Bolger AM, Lohse M, Usadel B. Trimmomatic: a flexible trimmer for Illumina sequence data. Bioinformatics. 2014;30(15):2114–20. doi: 10.1093/bioinformatics/btu170 24695404 PMC4103590

[16] Wick RR. Porechop: an adapter trimmer for Oxford Nanopore reads. 2018. https://github.com/rrwick/Porechop.

[17] Wick RR. Filtlong: quality filtering tool for long reads. 2021. GitHub—rrwick/Filtlong: quality filtering tool for long reads.

[18] Wick RR, Judd LM, Gorrie CL, Holt KE. Unicycler: Resolving bacterial genome assemblies from short and long sequencing reads. PLoS Comput Biol. 2017;13(6):e1005595. doi: 10.1371/journal.pcbi.1005595 28594827 PMC5481147

[19] Wick RR, Schultz MB, Zobel J, Holt KE. Bandage: interactive visualization of de novo genome assemblies. Bioinformatics. 2015;31(20):3350–2. doi: 10.1093/bioinformatics/btv383 26099265 PMC4595904

[20] Villesen P. FaBox: an online toolbox for fasta sequences. Mol Ecol Notes. 2007;7:965–968.

[21] Gurevich A, Saveliev V, Vyahhi N, Tesler G. QUAST: quality assessment tool for genome assemblies. Bioinformatics. 2013;29(8):1072–5. doi: 10.1093/bioinformatics/btt086 23422339 PMC3624806

[22] Seppey M, Manni M, Zdobnov EM. BUSCO: Assessing Genome Assembly and Annotation Completeness. Methods Mol Biol. 2019;1962:227–245. doi: 10.1007/978-1-4939-9173-0_14 31020564

[23] Walker BJ, Abeel T, Shea T, Priest M, Abouelliel A, Sakthikumar S, Cuomo CA, et al. Pilon: an integrated tool for comprehensive microbial variant detection and genome assembly improvement. PLoS One. 2014;9(11):e112963. doi: 10.1371/journal.pone.0112963 25409509 PMC4237348

[24] Seemann T. Prokka: rapid prokaryotic genome annotation. Bioinformatics. 2014;30(14):2068–9. doi: 10.1093/bioinformatics/btu153 24642063

[25] Chen L, Yang J, Yu J, Yao Z, Sun L, Shen Y, Jin Q. VFDB: a reference database for bacterial virulence factors. Nucleic Acids Res. 2005;33(Database issue):D325–8. doi: 10.1093/nar/gki008 15608208 PMC539962

[26] McArthur AG, Waglechner N, Nizam F, Yan A, Azad MA, Baylay AJ, Bhullar K, et al. The comprehensive antibiotic resistance database. Antimicrob Agents Chemother. 2013;57(7):3348–57. doi: 10.1128/AAC.00419-13 23650175 PMC3697360

[27] Cook A. The Mengo Hospital notes for the year 1897. Makerere College Medical School Library, Kampala, Uganda, 1897.

[28] MacCullum P. A new mycobacterial infection in man; clinical aspects. J Pathol Bacteriol. 1948;60(1):93–102. 18935206

[29] Anyim MC, Meka AO, Chukwu JN, Nwafor CC, Oshi DC, Madichie NO, et al. Secondary bacterial isolates from previously untreated Buruli ulcer lesions and their antibiotic susceptibility patterns in Southern Nigeria. Rev Soc Bras Med Trop. 2016;49(6):746–751. doi: 10.1590/0037-8682-0404-2016 28001222

[30] Smith K, Collier A, Townsend EM, O’Donnell LE, Bal AM, Butcher J, et al. One step closer to understanding the role of bacteria in diabetic foot ulcers: characterising the microbiome of ulcers. BMC Microbiol. 2016 Mar 22;16:54. doi: 10.1186/s12866-016-0665-z 27005417 PMC4804642

[31] Vandamme P, Heyndrickx M, Vancanneyt M, Hoste B, De Vos P, Falsen E, et al. *Bordetella trematum* sp. nov., isolated from wounds and ear infections in humans, and reassessment of *Alcaligenes denitrificans* Rüger and Tan 1983. Int J Syst Bacteriol. 1996;46(4):849–58. 8863408 10.1099/00207713-46-4-849

[32] Daxboeck F, Goerzer E, Apfalter P, Nehr M, Krause R. Isolation of *Bordetella trematum* from a diabetic leg ulcer. Diabet Med. 2004;21(11):1247–8. 15498093 10.1111/j.1464-5491.2004.01310.x

[33] Hernández-Porto M, Cuervo M, Miguel-Gómez MA, Delgado T, Lecuona M. *Bordetella trematum* como agente colonizador en úlcera de pie diabético. Rev Esp Quimioter. 2013;26(1):72–3. . 23546468

[34] Almagro-Molto M, Eder W, Schubert S. *Bordetella trematum* in chronic ulcers: report on two cases and review of the literature. Infection. 2015;43(4):489–94. doi: 10.1007/s15010-014-0717-y . 25600927

[35] Benthien S, Schlüter C, Becker SL, Papan C. Detection of *Bordetella trematum* in a diabetic patient with a skin and soft tissue infection. Int J Infect Dis. 2019;89:1–2. doi: 10.1016/j.ijid.2019.08.025 . 31472237

[36] Buechler C, Neidhöfer C, Hornung T, Neuenhoff M, Parčina M. Detection and Characterization of Clinical *Bordetella trematum* Isolates from Chronic Wounds. Pathogens. 2021;10(8):966. doi: 10.3390/pathogens10080966 . 34451430 PMC8401678

[37] Lacasse M, Inyambo K, Lemaignen A, Mennecart M, Gensburger S, Valentin AS, et al. Erysipelas of the right arm due to *Bordetella trematum*: a case report. J Med Case Rep. 2021;15(1):365. doi: 10.1186/s13256-021-02896-1 . 34253232 PMC8276433

[38] Majewski LL, Nogi M, Bankowski MJ, Chung HH. *Bordetella trematum* sepsis with shock in a diabetic patient with rapidly developing soft tissue infection. Diagn Microbiol Infect Dis. 2016;86(1):112–4. doi: 10.1016/j.diagmicrobio.2016.05.019 . 27397578

[39] Castro TR, Martins RCR, Dal Forno NLF, Santana L, Rossi F, Schwarzbold AV, et al. *Bordetella trematum* infection: case report and review of previous cases. BMC Infect Dis. 2019;19(1):485. doi: 10.1186/s12879-019-4046-8 . 31146691 PMC6543606

[40] Almuzara M, Barberis C, Traglia G, Sly G, Procopio A, Vilches V, et al. Isolation of *Bordetella* species from unusual infection sites. JMM Case Reports. 2015;2(2). doi: 10.1099/jmmcr.0.000029

[41] Kitagawa D, Kurimoto T, Oyama S, Suzuki S, Masuo K. A case of *Bordetella trematum* and Kerstersia gyiorum infections in a patient with congestive dermatitis. J Infect Chemother. 2021;27(5):740–746. doi: 10.1016/j.jiac.2020.12.008 . 33386260

[42] Smith JL, Bayles DO. The contribution of cytolethal distending toxin to bacterial pathogenesis. Crit Rev Microbiol. 2006;32(4):227–48. doi: 10.1080/10408410601023557 . 17123907

[43] Wising C, Mölne L, Jonsson I-M, Ahlman K, Lagergård T. The cytolethal distending toxin of *Haemophilus ducreyi* aggravates dermal lesions in a rabbit model of chancroid. Microbes and Infection. 2005;7: 867–874. doi: 10.1016/j.micinf.2005.02.009 15876546

[44] Hammoudi N, Verdot R, Delorme J, Bouam A, Drancourt M. Screening anti-infectious molecules against *Mycobacterium ulcerans*: A step towards decontaminating environmental specimens. PLOS ONE. 2020;15: e0231685. doi: 10.1371/journal.pone.0231685 . 32760069 PMC7410233

[45] Halim I, Ihbibane F, Belabbes H, Zerouali K, El Mdaghri N. Isolement de Bordetella trematum au décours d’une bactériémie. Ann Biol Clin (Paris). 2014;72(5):612–4. doi: 10.1684/abc.2014.0998 . 25336136

[46] Chauty A, Ardant M-F, Adeye A, Euverte H, Guédénon A, Johnson C, et al. Promising Clinical Efficacy of Streptomycin-Rifampin Combination for Treatment of Buruli ulcer. Antimicrob. Agents Chemother. 2007;51: 4029–4035. doi: 10.1128/AAC.00175-07 17526760 PMC2151409

[47] Klis S, Stienstra Y, Phillips RO, Abass KM, Tuah W, Van Der Werf TS. Long Term Streptomycin Toxicity in the Treatment of Buruli Ulcer: Follow-up of Participants in the BURULICO Drug Trial. PLOS Neglected Tropical Diseases. 2014;8: e2739. doi: 10.1371/journal.pntd.0002739 24625583 PMC3953024

[48] Adriaans B, Hay R, Lucas S, Robinson DC. Light and electron microscopic features of tropical ulcer. J Clin Pathol. 1987;40(10):1231–4. doi: 10.1136/jcp.40.10.1231 . 3680548 PMC1141201

[49] Griesenauer B, Xing Y, Fortney KR, Gao X, González-Beiras C, Nelson DE, et al. Two Streptococcus pyogenes emm types and several anaerobic bacterial species are associated with idiopathic cutaneous ulcers in children after community-based mass treatment with azithromycin. PLOS Neglected Tropical Diseases. 2022;16: e0011009. doi: 10.1371/journal.pntd.0011009 36534698 PMC9810193

