# Supplementary material for: Mycobacterium ulcerans-Bordetella trematum chronic tropical cutaneous ulcer: A four-case series, Côte d’Ivoire
Source: PLoS Negl Trop Dis. 2023 Dec 7;17(12):e0011413. doi: 10.1371/journal.pntd.0011413 (PMC10703317; doi:10.1371/journal.pntd.0011413)
Supplement: S1 Text — (DOCX) [file pntd.0011413.s001.docx]

Supplementary data.


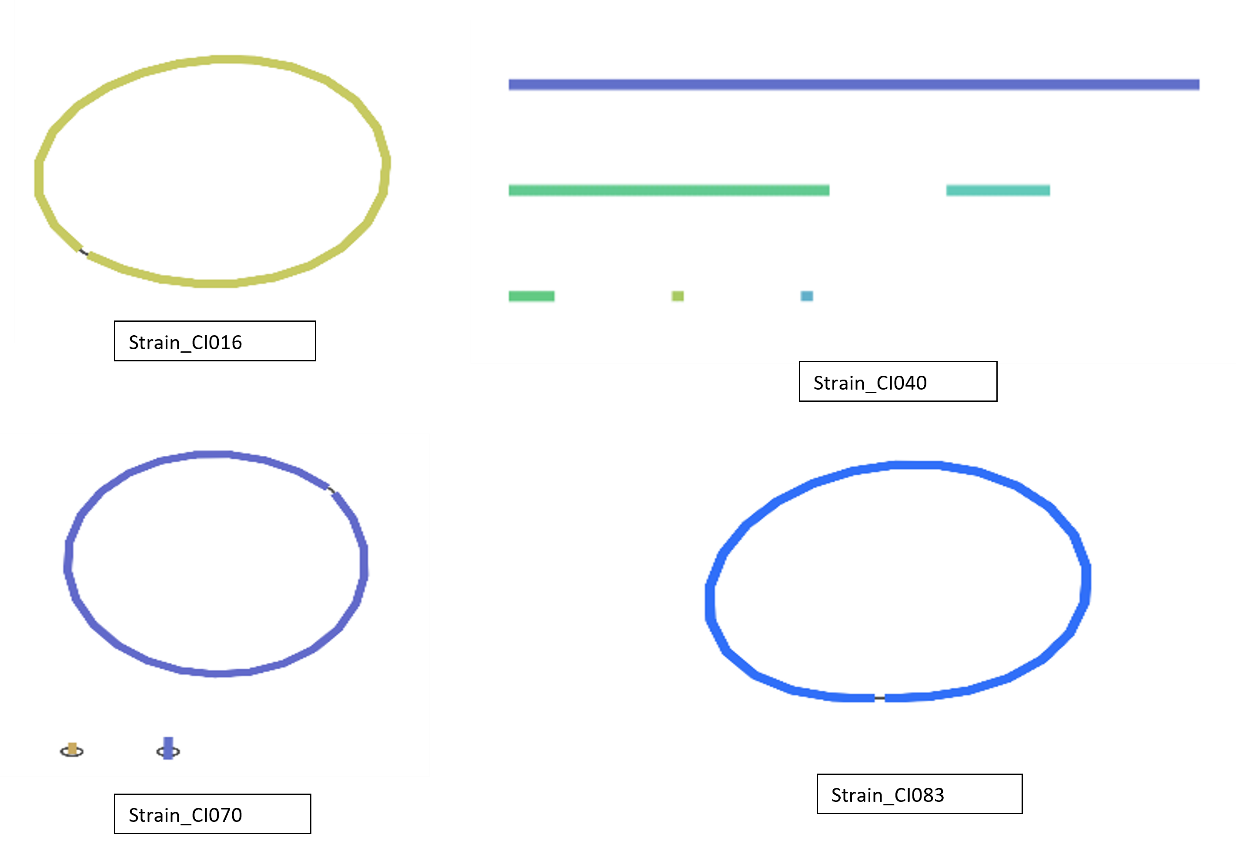


**Fig A:** *De novo* assembly graph of hybrid assembly of four *Bordetella trematum* strains’ genomes.

**Table A**: *Bordetella trematum* strains’ genome assembly, contiguity and completeness.

|  | Metric | **Strain_CI016** | **Strain_CI040** | **Strain_CI070** | **Strain_CI083** | **Strain_CI016** | **Strain_CI040** | **Strain_CI070** | **Strain_CI083** |
| --- | --- | --- | --- | --- | --- | --- | --- | --- | --- |
| Contiguity | Contigs | 1 | 6 | 3 | 1 | 1 | 1 | 3 | 1 |
|  | Total length | 4397893 | 4541708 | 4342488 | 4427276 | 4397920 | 4541719 | 43424665 | 4427308 |
|  | GC (%) | 65.58 | 65.60 | 65.59 | 65.62 | 65.58 | 65.60 | 65.59 | 65.62 |
|  | N50 | 4397893 | 2673161 | 4342488 | 4427276 | 4397920 | 4541719 | 43424665 | 4427308 |
| Completeness (569 cores genes (BUSCO)) | Fragmented genes | 2 | 2 | 3 | 5 | 2 | 2 | 3 | 2 |
|  | Intact genes | 564 | 565 | 563 | 562 | 565 | 565 | 563 | 565 |
|  | Lost genes | 3 | 2 | 3 | 2 | 2 | 2 | 3 | 2 |
|  | Completeness score (strict, %) | 99,1 | 99,3 | 98,9 | 98,7 | 99,3 | 99,3 | 98,9 | 99,3 |

**Preliminary assembly**

**Final assembly**
